# Median effective effect-site concentration of sufentanil for wake-up test in adolescents undergoing surgery: a randomized trial

**DOI:** 10.1186/s12871-015-0003-2

**Published:** 2015-03-08

**Authors:** Cheng-Hua Zhang, Wei-Qing Ma, Yun-Li Yang, Hui-Ming Wang, Fa-Tuan Dong, Zhang-Xiang Huang

**Affiliations:** Department of Anesthesiology, Kunming General Hospital of Chengdu Military Area, Kunming, 650032 China

**Keywords:** Sufentanil, Idiopathic scoliosis, Intraoperative wake-up test

## Abstract

**Background:**

To determine the median effective concentration of sufentanil as an analgesic during wake-up tests after sevoflurane anesthesia during surgery for adolescent idiopathic scoliosis (AIS).

**Methods:**

This is a randomised controlled trial. Sixty patients aged 13–18 years scheduled for AIS surgery were randomized into six groups of 10 patients each to receive target effect-site concentrations of sufentanil of 0.19, 0.1809, 0.1723, 0.1641, 0.1563, and 0.1489 ng/ml (target concentration ratio, 1.05). Wake-up time was recorded. Median EC_50_ and 95% confidence interval (CI) for sufentanil target-controlled infusion (TCI) were determined using Kärber’s method. The primary outcome was median EC_50_ for sufentanil TCI as an analgesic during the wake-up test after sevoflurane anesthesia during surgery for AIS.

**Results:**

The EC_50_ and 95% CI of sufentanil TCI were 0.1682 ng/ml and 0.1641 ~ 0.1724 ng/ml, respectively.

**Conclusions:**

The EC_50_ of sufentanil TCI was 0.1682 ng/ml (95% CI: 0.1641 ~ 0.1724 ng/ml) during sevoflurane anesthesia in adolescents undergoing surgery for idiopathic scoliosis with intraoperative wake-up tests.

**Trial registration:**

Clinicaltrials.gov identifier: ChiCTR-TTRCC-12002696.

## Background

Adolescent idiopathic scoliosis (AIS) is one of the most common structural spinal deformities in the coronal plane affecting young people. Depending on severity, treatment consists mainly of observation, braces and surgical correction [1], with surgical correction required only when the deformity is greater than 40° [2]. The most important neurological complication during surgery for AIS is iatrogenic spinal cord injury. The wake-up test is the standard neurophysiological procedure for the intraoperative detection of emerging spinal cord injuries during the surgical correction of AIS [3]. In addition to faster recovery of consciousness from anesthesia, the wake-up test is accompanied by several adverse phenomena, including coughing, agitation, hypertension, and tachycardia [4]. Thus, during surgery for AIS, the anesthesiologist must balance adequate sedation and analgesia to prevent bad memories as a result of postoperative recall and sufficient consciousness to respond to commands [5].

Recent reports have indicated that target-controlled infusion (TCI) of sufentanil is more useful for achieving analgesia and preventing cough and agitation [6] than remifentanil, a short-action opioid [7,8] during anesthesia. Balanced anesthesia is usually achieved by the coadministration of sufentanil, a long-acting opioid, and sevoflurane [9], with the two agents acting synergetically to reduce the adverse effects of each as a single agent.

To our knowledge, however, no study has investigated the median effective concentration of sufentanil as an analgesic during the wake-up test after sevoflurane anesthesia during surgery for AIS. We therefore determined the median effective concentration of sufentanil required to provide excellent analgesic effects in these patients after sevoflurane anesthesia.

## Methods

Following approval by the ethics committee of Kunming General Hospital of the Chengdu Military Area (Trial registration number: ChiCTR-TTRCC-12002696) and written, informed consent from the patients and their parents, we enrolled consecutive ASA physical status I patients, aged 13–18 years, scheduled to undergo surgery for AIS. Sixty patients in this double-blinded study were randomized to 6 groups (n = 10) according to a table of random numbers, since 5 ~ 20 patients per group are thought sufficient for the Behrens-Kärber method. The target effect-site concentration of each group during wake-up tests was defined as the preliminary result; beginning with 0.19 ng/ml, the concentration ratio was 1.05. Exclusion criteria included history of neurological, cardiac or pulmonary disease, any central nervous system disease, long-term administration of sedatives or a history of alcohol abuse. The day before surgery, all enrolled patients received detailed instructions about the wake-up test.

### Anesthetic preparation

Perioperative monitoring included 5-lead electrocardiogram (ECG), pulse oxygen saturation (SpO_2_), heart rate (HR), central venous pressure (CVP), and invasive blood pressure (mean artery pressure, MAP). A bispectral index (BIS) monitor (BIS-XP monitor, Aspect Medical Systems Inc., Norwood, MA, USA) was applied to each patient. Muscle relaxation level was monitored with TOF-Watch® SX (Organon, The Netherlands).

The TCI pump administered a target effect-site concentrations of sufentanil (sufentanil Gepts model) based on t_1/2_, K_eo_ and patient age. Before induction of anesthesia, the TCI of sufentanil as a co-adjuvant analgesic was started with a target Ce of 0.5 ng/ml, based on a preliminary experiment, and intravenous etomidate was administrated at a dose of 0.15 ~ 0.3 mg/kg. Once the patient lost consciousness, cisatracurium besylate 0.15 mg/kg was administered iv as a muscle relaxant. Thereafter, the trachea was intubated and the lungs were mechanically ventilated.

Expired concentrations of sevoflurane, carbon dioxide (CO_2_), and oxygen were measured continuously using the anesthesia workstation (Zeus®Infinity Empowered, Drager Medical, Lubeck, Germany), with the end-tidal concentration of sevoflurane maintained between 0.8% ~ 1.5% and end tidal CO_2_ between 30 ~ 40 mmHg (1 mmHg = 0.133 kPa). Paralysis was maintained with a continuous infusion of cisatracurium besylate 0.1 mg/kg/h and a TCI of sufentanil (0.2 ~ 0.3 ng/ml) using a TCI-III pump (Orchestra® Base Primea, Fresenius Vial, France). Sufentanil dose was based on a preliminary test. Adequate anesthesia was defined as a BIS value between 40 and 55 and maintenance of HR and MAP without exceeding 20% of the baseline value.

### Wake-up test

During this period, no cisatracurium besylate was injected, infusion pumps were stopped, the end-tidal concentration of sevoflurane was 0, and the target concentrations of sufentanil in all groups were down-regulated to the target effect-site concentrations of 0.19, 0.1809, 0.1723, 0.1641, 0.1563, and 0.1489 ng/ml (target concentration ratio 1.05). Five minutes later, the patient’s name was repeated every 15 seconds, followed by a request to move both feet. If the patients did not respond to command within 15 min, the TCI of sufentanil was stopped until the patients responded. When the wake-up test was completed, cisatracurium besylate 0.03 mg/kg was administrated i.v. and the infusion pump was restarted. The lungs were ventilated again and anesthesia was maintained in each group as before. Within 24 hours after surgery, vital signs, consciousness and intraoperative awareness were determined in all patients once every 4 hours.

The patient’s name was repeated every 15 seconds, followed by a request to move both feet. Success was defined as a response to command within 15 min. The numbers of successful cases (r) were recorded in each group and the logarithm of target effect-site concentration (x), success (p) and failure (q) rates of the wake-up test (p), the logarithm of maximum target effect concentration (*x*_*m*_) and the difference in the logarithm of adjacent concentrations (*i*) were calculated. The data were also evaluated according to the Behrens-Kärber method [10].

$$ {\mathrm{EC}}_{50} = { \lg}^{-1}\left[{\chi}_m-i\left({\displaystyle \sum p-0.5}\right)\right] $$

$$ {\mathrm{S}}_{{\mathrm{lgEC}}_{50}}=i\sqrt{{\displaystyle \sum \frac{pq}{n}}} $$

$$ 95\%\mathrm{C}\mathrm{I}:\ \left[{ \lg}^{-1}\left({\mathrm{lgEC}}_{50}-1.96\ {\mathrm{S}}_{{\mathrm{lgEC}}_{50}}\right),\ { \lg}^{-1}\left({\mathrm{lgEC}}_{50} + 1.96\ {\mathrm{S}}_{{\mathrm{lgEC}}_{50}}\right)\right] $$

### Statistics

Statistical analysis was performed using SPSS 17.0 for Windows. Data are expressed as means ± sd and analyzed using one-way ANOVA. Categorical data were expressed as the number of patients (%) and analyzed using the *χ*^2^ test. A P-value <0.05 was considered statistically significant.

## Results

Sixty patients successfully completed the study. The 6 patient groups were similar in age, body mass index (BMI), gender and ASA distribution (Table 1). Duration from induction of anesthesia to start of the wake-up test did not differ significantly among the patient groups.

**Table 1:** **Characteristics of patient groups**

Group	Sex (M/F)	Age (year)	BMI (kg/m^2^)	Anesthesia duration before wake up (min)
1	5/5	15 ± 3	21 ± 2	235 ± 36
2	6/4	14 ± 3	22 ± 2	228 ± 44
3	4/6	14 ± 4	22 ± 2	241 ± 34
4	4/6	15 ± 3	21 ± 3	246 ± 30
5	5/5	15 ± 3	22 ± 2	239 ± 38
6	6/4	14 ± 3	21 ± 2	242 ± 37

n = 10 per group. Results reported as mean ± sd.

Two patients had adverse reactions during operation, with one having hypertension and tachycardia and one having cough/restless reaction. None of these patients experienced bad memories.

The sufentanil EC_50_ as an analgesic during sevoflurane anesthesia was 0.1682 ng/ml (95% CI, 0.1641 ~ 0.1724 ng/ml) (Table 2).

**Table 2:** **Success and failure rate in each group and EC**_**50**_ **(95%CI) results**

Group	Target effect-site concentration of sufentanil (ng/ml)	Logarithm of target effect-site concentration (x)	Numbers of successful (r)	Success rate (p) (%)	Failure rate (q) (%)	EC_50_(95%CI) (ng/ml)
1	0.1900	−0.7212	0	0	100	0.1682 (0.1641 ~ 0.1724)
2	0.1809	−0.7424	1	10	90	
3	0.1723	−0.7636	4	40	60	
4	0.1641	−0.7848	6	60	40	
5	0.1563	−0.8060	9	90	10	
6	0.1489	−0.8272	10	100	0	

95%CI: 95% confidence interval.

## Discussion

Neurologic deficits may be caused directly by surgery for AIS [11]. Once complete spinal cord damage has occurred, the likelihood of recovery is limited. Use of intraoperative wake-up tests may decrease the incidence of over- or undercorrection, preventing the progression of spinal cord injury [12]. During the wake-up procedure, the depth of anesthesia should be lightened so that patients respond to the surgeon’s verbal commands. Recovery of awareness, however, may be accompanied by coughing and agitation resulting from the operation itself. Although sufentanil is an analgesic, not a hypnotic, it has been found to affect opioid receptors in the limbic system, eliminating the emotional reactions induced by pain, such as anxiety, tension, and inability to easily fall asleep. Sufentanil may dose-dependently enhance the inhibitory effect of sevoflurane on the CNS, with a high concentration of sufentanil affecting awakening speed and enhancing the effect of anesthetics [13]. TCI sufentanil model parameters have been validated for this patient population [14]. Sufentanil has been found to result in better analgesia and more stable hemodynamics [15], and the combination of sufentanil and the inhaled anesthetic sevoflurane has been found to produce synergistic effects in the induction of anesthesia [16].Sufentanil has been found to improve the quality of awakening [15] and to reduce the likelihood of postoperative complications [17]. Of our 60 patients, none recalled intraoperative pain or other events after the wake-up test, a result likely due to their treatment with combined anesthesia (sevoflurane and sufentanil) rather than one drug [18].

Ideally, adequate analgesia should be accompanied by a lack of effect on the recovery of consciousness. To minimize the effect-site concentration of sufentanil under the wake-up test during surgery for AIS, we decreased the end-tidal concentration of sevoflurane to 0 to eliminate the effect of sevoflurane on the faster recovery of consciousness [19] and calculated the median effective concentration of sufentanil by the Behrens-Kärber method. Our preliminary results indicated that patients did not respond to command when the target effect-site concentration was 0.19 ng/ml, but did respond when the target effect-site concentration was 0.14 ng/ml. Therefore, using the Behrens-Kärber method, the six indicated effect site concentrations were chosen. We found that the minimum effect-site concentration of sufentanil was 0.14 ng/ml and the EC_50_ of sufentanil TCI was 0.1682 ng/ml (95% CI: 0.1641 ~ 0.1724 ng/ml). Induction of anesthesia with a combination of sevoflurane and sufentanil may improve the success rate of intraoperative wake-up tests. These tests should measure both the appropriate depth of sedation analgesia that effectively inhibits nociceptive stimuli caused by stress and the rate of successful awakening. A higher effect-site concentration of sufentanil, within its appropriate range, may inhibit stress responses induced by nociceptive stimuli such as the insertion of an endotracheal tube and surgical incision, due to a stronger analgesic effect. Higher doses, outside the appropriate range, may cause delay or failure of wake-up tests, whereas doses lower than the appropriate range may result in stronger stress responses. We observed a sufentanil EC_50_ of 0.1682 ng/ml, making the lower limit of the appropriate range 0.14 ng/ml, a concentration providing a reference for appropriate depth of clinical analgesia.

A major shortcoming of this study was our lack of measurement of sufentanil concentrations. However, good correlations have been observed among target effect-site concentrations, plasma concentrations and clinical indicators, including blood pressure, heart rate and BIS [20,21]. Therefore, any errors due to interindividual variability and differences between target effect-site concentrations and plasma concentrations were likely negligible.

## Conclusion

In conclusion, we found that the inhalation of sevoflurane combined with sufentanil TCI resulted in uncomplicated induction of anesthesia. Furthermore, we showed that the EC_50_ of the sufentanil effect-site concentration for analgesia during sevoflurane anesthesia in surgery for AIS with an intraoperative wake-up test was 0.1682 ng/ml (95% CI, 0.1641 ~ 0.1724 ng/ml). Anesthesia induction with the combination of sevoflurane and sufentanil may improve the success rate of intraoperative wake-up tests.
